# Three-dimensional computed tomography reconstruction of the gastroesophageal junction following hiatal hernia repair with Collis gastroplasty and fundoplication: a novel method to demonstrate postoperative morphology and better understand hiatal hernia recurrence

**DOI:** 10.1590/0102-67202026000006e1935

**Published:** 2026-06-22

**Authors:** Renato Abrantes LUNA, Vitor Moreira SARDENBERG, Romulo Varella de OLIVEIRA, Gerson da Silva RIBEIRO, Maria Eduarda Azevedo LIMA, Heron Werner, Daiane DECKER, John G. HUNTER

**Affiliations:** 1 Hospital Federal dos Servidores do Estado do Rio de Janeiro, Department of General Surgery – Rio de Janeiro (RJ), Brazil.; 2 Pontifícia Universidade Católica, Radiology Department – Rio de Janeiro (RJ), Brazil.; 3 Universidade Estadual do Estado do Rio de Janeiro, Radiology Department – Rio de Janeiro (RJ), Brazil.; 4 Pontifícia Universidade Católica, DASA Biodesign Laboratory, Department of Fetal Medicine – Rio de Janeiro (RJ), Brazil.; 5 Secretaria Municipal de Saúde, Cypriano das Chagas Medeiros Family Clinic – Rio de Janeiro (RJ), Brazil.; 6 Oregon Health & Science University, Department of Surgery, Division of Gastrointestinal and General Surgery – Portland, USA.

**Keywords:** Gastroplasty, Hernia, Hiatal, Fundoplication, Gastroesophageal junction, Tomography, Gastroplastia, Hérnia hiatal, Fundoplicatura, Junção esofagogástrica, Tomografia

## Abstract

**Background:**

Collis gastroplasty for esophageal lengthening is a complex adjunct to hiatal hernia repair in patients with esophageal foreshortening.

**Aims:**

To study the final morphology of the repair using state-of-the-art imaging: computed tomography with three-dimensional reconstruction.

**Methods:**

Nine patients with prior Collis gastroplasty and hiatal hernia repair were studied with three-dimensional computed tomography reconstruction to evaluate the anatomy of the repair and screen for hiatal hernia recurrence. Secondary outcomes were quality of life and surgical morbidity.

**Results:**

After a medium follow-up of 34 months, objective recurrence of the hiatal hernia was observed in three patients (1.5, 2.2, and 3 cm), and two patients were symptomatic. The gastroesophageal junction tube (neo-esophagus) created by the gastroplasty was similar in shape and volume to the native esophagus in all patients. The fundoplication previously performed covered the neo-esophagus in only two of the nine patients. No fistulas or mortality were observed.

**Conclusions:**

Three-dimensional computed tomography reconstruction of the gastroesophageal junction following hiatal hernia repair with Collis gastroplasty and fundoplication reliably demonstrates postoperative anatomy and helps better understand hiatal hernia recurrence.

## ARTICLE HIGHLIGHTS

Collis gastroplasty is an advanced technique used during hiatal hernia repair when the gastroesophageal junction cannot be positioned at least 3 cm below the diaphragm.

It is typically indicated in patients with giant hiatal hernias, redo operations, Barrett’s esophagus, or peptic strictures who are most likely to have esophageal foreshortening.

Three-dimensional computed tomography reconstruction provides a detailed analysis of Collis gastroplasty morphology, demonstrating a uniform neo-esophagus and fundoplication.

## Summary

**CENTRAL MESSAGE:**

Collis gastroplasty is an advanced surgical technique used during hiatal hernia repair when the gastroesophageal junction cannot be positioned at least 3 cm below the diaphragm. Its primary purpose is to lengthen the esophagus by tubularizing 3 cm or more of the gastric cardia (neoesophagus) to reduce the axial tension of the repair and decrease hiatal hernia recurrence rates. The procedure is typically indicated in patients with giant hiatal hernias, redo operations, Barrett’s esophagus, or peptic strictures who are most likely to have esophageal foreshortening.

**PERSPECTIVES:**

Collis gastroplasty reliably produces a tubular gastric conduit resembling the native esophagus in shape and volume. Three-dimensional computed tomography is a promising tool for evaluating the postoperative morphology of the repair and anatomical recurrences, demonstrating the durable anatomical structure of Collis gastroplasty.

## VISUAL ABSTRACT

**Figure:**
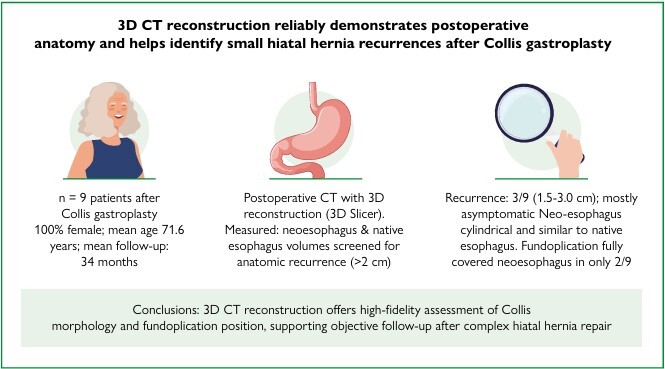

## RESUMO

**Racional:**

A gastroplastia de Collis para alongamento esofágico é um procedimento complexo, complementar à correção de hérnia hiatal, em pacientes com encurtamento esofágico.

**Objetivos:**

Estudar a morfologia final da correção utilizando tomografia computadorizada (TC) com reconstrução tridimensional (3D).

**Métodos:**

Nove pacientes submetidos previamente a gastroplastia de Collis e correção de hérnia hiatal foram estudados com reconstrução 3D por TC para avaliar a anatomia da correção e avaliar recidivas da hérnia hiatal. Os desfechos secundários foram a qualidade de vida e a morbidade cirúrgica.

**Resultados:**

Após um acompanhamento médio de 34 meses, foi observado recidiva objetiva da hérnia hiatal em 3 pacientes (1,5, 2,2 e 3 cm) e dois pacientes apresentaram sintomas. O tubo da junção gastroesofágica (neoesôfago) criado pela gastroplastia apresentou forma e volume semelhantes ao esôfago nativo, em todos os pacientes. A fundoplicatura realizada anteriormente cobriu o neoesôfago em apenas 2 dos 9 pacientes. Não foram observadas fístulas ou mortalidade.

**Conclusões:**

A reconstrução tridimensional por tomografia computadorizada da junção gastroesofágica após reparo de hérnia hiatal com gastroplastia de Collis e fundoplicatura demonstra de forma confiável a anatomia pós-operatória e auxilia na melhor compreensão da recorrência da hérnia hiatal.

## INTRODUCTION

 Collis gastroplasty is an advanced surgical technique used during hiatal hernia repair when the gastroesophageal junction (GEJ) cannot be positioned at least 3 cm below the diaphragm. Its primary purpose is lengthening the esophagus by tubularizing 3 cm or more of the gastric cardia (neo-esophagus) to reduce the axial tension of the repair and decrease hiatal hernia recurrence rates^4,9^. The procedure is typically indicated in patients with giant hiatal hernias, redo operations, Barrett’s esophagus, or peptic strictures who are most likely to have esophageal foreshortening^1,5^. 

 This study aims to evaluate the final anatomical configuration of Collis gastroplasty using state-of-the-art imaging — computed tomography (CT) with three-dimensional (3D) reconstruction. Secondary outcomes include quality of life, perioperative complications, and symptomatic recurrence rates. 

## METHODS

 We conducted a retrospective case series of patients who underwent Collis gastroplasty performed by a single surgeon (RAL). Patients were invited for postoperative CT imaging, whether symptomatic or not, to evaluate Collis morphology and hiatal hernia recurrence. This study was approved by a local Ethics Committee (71318223.0.0000.5533) according to the Declaration of Helsinki. All patients signed an informed consent form before the radiologic study. The authors have no financial support from industry nor conflict of interest. 

 As a part of a laparoscopic hiatal hernia, a wedge-type Collis gastroplasty was performed when the tension-free intra-abdominal esophageal length was <2.5 cm. The gastroplasty was accomplished by positioning a 48-56 French bougie at the GEJ and firing sequential 45 mm stapler loads (purple cartridge) from the greater curvature toward the bougie, to a point 3 cm below the GEJ. Further stapler firings towards the angle of His created a tubularized gastric conduit ("neo-esophagus")^8^. The vertical staple line was oversewn using 3-0 polypropylene suture. In one case, a 32 French Fouchet tube was used. 

 Following esophageal lengthening and hiatal closure, patients underwent a fundoplication: a 180° Toupet (target valve length 3 cm) or a 360° Nissen (target 2 cm, floppy valve)^6^. Mesh reinforcement of the hiatal repair and gastropexy were added at the surgeon’s discretion. Postoperative complications classified as Clavien–Dindo ≥II were recorded^2^. 

 Postoperative CT images were obtained using 160 or 320 channel scanners with 0.5 mm collimation after a >4-hour fast. Imaging was done in the supine position. After an initial acquisition, oral iodinated contrast (iohexol 300 mg I/mL, diluted 20 mL in 200 mL water) was administered, followed by intravenous contrast (iobitridol 350 mg I/mL) at a dose of 1–2 mL/kg at 3–4 mL/s. Arterial (35 sec) and portal venous (70 sec) phases were captured. 

**Figure 1:**
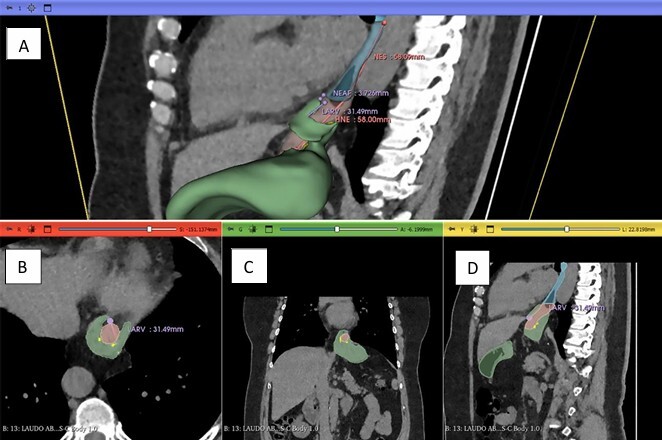
Segmentation and 3D reconstruction (case 1, patient without recurrence). (A) Merge between 3D reconstruction and sagittal plane; (B) Axial plane; (C) Coronal plane; (D) Sagittal plane. Four images are simultaneously displayed for accurate measurements.

 Three-dimensional reconstructions were performed using the 3D Slicer software after importing DICOM files into the software^3^. The first step was image segmentation, where the designer defined the structures in each slice of the CT scan. After the first raw definition, the segmentation was refined together with the surgeon. Once the segmentation was finished, the 3D reconstruction was performed. The measurements were done with CT images in the coronal, axial, and sagittal planes, along with the 3D image, allowing for a more precise definition (Figure 1). 

 For purposes of comparison, a length of native tubular esophagus (NTS) immediately above and equivalent in length to the neo-esophagus was defined. We also measured the height of the neo-esophagus above the fundoplication (NEAF). CT evaluation included recurrence, configuration, volumes of the neo-esophagus and the native esophagus, and the length of the anti-reflux valve. 

 Volume calculations were done using 3D software and confirmed with the cylindrical volume formula. An independent board-certified radiologist reviewed all scans for recurrence. Quality of life was assessed using the validated Gastroesophageal Reflux Disease-Health Related Quality of Life (GERD-HRQL) questionnaire^10^. 

## RESULTS

 Among 96 anti-reflux procedures performed during the study period, 12 (13%) patients were found to have a foreshortened esophagus and underwent Collis gastroplasty. Of these, nine patients underwent postoperative 3D CT imaging. 

 All nine patients were female, with a mean age of 71.6 years (range: 57.8–81.2), a mean body mass index (BMI) of 31.09 kg/m^2^ (27.5–33), and a mean follow-up of 34.3 months (range: 0.9–72.5). The average length of stay was 3,56 days (2 to 7). Four patients presented with obstructive symptoms (dysphagia, bloating, nausea, vomiting, and chest pain); five had persistent heartburn and regurgitation despite lifestyle changes and medications. One patient with obstructive symptoms also had gastric volvulus. 

 Hiatal hernia types included eight Type III and one Type I (>4 cm). Six patients received Toupet fundoplication, and three patients underwent Nissen fundoplication. Mesh was placed in five cases, and gastropexy was used in four (including one tube gastrostomy). All procedures were completed laparoscopically with no conversions. Mean hiatal surface area was 9.75 cm² (3.5–15 cm^2^). In most patients, the neo-esophagus was longer than the fundoplication, and only in two patients did the valve completely cover the neo-esophagus. 

 No fistulas or leaks were observed. One patient was reoperated on postoperative day one for early acute herniation, the result of immediate postoperative retching. One patient had postoperative dysphagia, and one patient had postoperative diarrhea. In the patient who needed an early reoperation, the crural closure appeared inadequate, and the posterior gastric fundus was redundant. At the index operation, the patient had no mesh, and it was added at the reoperation. The Toupet was rebuilt with less redundant fundus, and an anterior and a posterior gastropexy were added. The patient recovered well without other complications. For the patient with dysphagia, two endoscopic dilations were needed with full symptom resolution. This patient had her Collis done over a 32-French Fouchet tube. The patient with persistent diarrhea was diagnosed with pancreatic insufficiency. 

 All patients reported satisfaction with the procedure, with a mean GERD-HRQL score of 6.8 (median: 3). Only two patients had scores >20 and resumed proton-pump inhibitor therapy. 

 Objective hiatal hernia recurrence was noted in three patients with 3D CT reconstruction (1.5 cm, 2.2 cm, and 3.0 cm). The neo-esophagus appeared cylindrical in all cases, except one, which had a conical shape (also the dysphagia case). Mean neo-esophagus and native esophagus segment volumes were 9.8 cm^3^ and 12.16 cm^3^, respectively. In one case, the valve length was shorter than planned (12 mm in a Toupet fundoplication). All fundoplication types were correctly identified on 3D CT. The summary of patients’ characteristics and 3D CT measurements are detailed in Table 1 and Table 2. 

**Table 1:** Patients’ characteristics and surgical outcomes (n=9).

Variable	Value
Age, years (mean±SD)	71.6±6.8
Female sex, n (%)	9 (100%)
BMI, kg/m^2^ (mean±SD)	31.1±1.7
Follow-up duration, months (mean)	34.3 (range: 0.9–72.5)
Hiatal hernia type, n (%)	Type I: 1 (11%) Type III: 8 (89%)
Fundoplication type, n (%)	Toupet: 6 (67%) Nissen: 3 (33%)
Mesh reinforcement, n (%)	5 (56%)
Gastropexy performed, n (%)	4 (44%)
Hiatal surface area, cm^2^ (mean)	9.75 (range: 3.5–15)
Complication (Clavien–Dindo ≥II), n (%)	3 (33%)
Postoperative mortality	0

SD: standard deviation; BMI: body mass index.

**Table 2:** Computed tomography-based anatomical measurements and recurrence findings.

Patient	Neo-esophagus volume (cm^3^)	Native esophagus volume (cm^3^)	Valve length (mm)	Fundoplication type	Recurrence (Y/N)	Conduit shape	GERD HRQL
1	9.078	9.21	33.16	Toupet	Y (3 cm)	Cylindrical	2
2	9.29	12.48	36.2	Nissen	Y (1.5 cm)	Cylindrical	20
3	7.81	8.145	22.94	Toupet	N	Cylindrical	22
4	14.78	13.27	31.49	Nissen	y (2.2 cm)	Cylindrical	3
5	8.82	13.26	12.3	Toupet	N	Conical	5
6	10.58	6.19	18.07	Nissen	N	Cylindrical	3
7	11.62	15.28	30.98	Toupet	N	Cylindrical	3
8	7.47	9.13	27.08	Toupet	N	Cylindrical	0
9	9.13	22.51	38.64	Toupet	N	Cylindrical	4

Notes: Valve length was measured via 3D reconstruction. Recurrence is defined as >2 cm herniation of gastric tissue above the crura. A conical shape was observed in the patient who experienced postoperative dysphagia.

GERD: Gastroesophageal Reflux Disease; HRQL: Health-Related Quality of Life.

## DISCUSSION

 This study is, to our knowledge, the first to evaluate laparoscopic Collis gastroplasty morphology using 3D CT reconstruction. With this imaging technique, anatomic details can be obtained that are difficult to obtain with fluoroscopic studies. For example, a conventional esophagram poorly differentiates between the native esophagus and the neo-esophagus. In this study, all neo-esophagi were tubular and similar in size and shape to the native esophagus. No distortion or deviation from the esophageal axis was noted. 

 The absence of mortality and low complication rates support the safety of this approach. One patient required reoperation, another had dysphagia likely due to a smaller bougie, and one developed non-surgical diarrhea. Importantly, no fistulas were observed, though prior studies report rates of 1.5%^5^. 

 Recurrences were anatomically minor and mostly asymptomatic, consistent with prior data showing the limited clinical significance of small anatomical recurrences^7^. 

 We also tried to address the portion of the neo-esophagus not covered by the fundoplication, since experts constantly reinforce this technical detail. It could be achieved in only two patients. One of them had a 3 cm anatomical hiatal hernia recurrence, but no symptoms, and the other had a 1,5 cm anatomical hiatal hernia recurrence with heartburn. In both cases, the fundoplication was intact. 

 The patient with early recurrence had a wide crural aperture associated with a fundoplication built with a redundant fundus and a gastropexy that was too low on the anterior gastric wall. Of course, the postoperative retching could be implicated, but it is always difficult to address whether the retching caused the early hiatus hernia recurrence or was a consequence of early fundoplication migration. This patient had the hiatus recalibrated, a mesh added for reinforcement, the fundoplication rebuilt, and the gastropexy repositioned more proximally. After 3.5 years of follow-up, she remains symptom-free and recurrence-free. For the patient with persistent dysphagia, it was attributed to the use of a smaller bougie. Thankfully, a couple of balloon dilations were enough to address the problem. This patient is doing well, without symptoms and recurrence-free, after almost 3.5 years. The third patient had diarrhea secondary to pancreatic insufficiency, which was being controlled with pancreatic enzymes. She has no symptoms or recurrences attributed to the hiatal hernia after 1.7 years. 

 The main strength of this study is the use of advanced 3D imaging to objectively evaluate postoperative anatomy. This technology enabled the assessment of conduit volume (the native esophagus and the neo-esophagus), morphology, and fundoplication characteristics in high fidelity. Mid- to long-term follow-up suggests durable anatomical results without dilation of the neo-esophagus. 

 Limitations of this study include the absence of postoperative pH monitoring, which limits conclusions about functional reflux control. Although small case series demonstrated a 50% rate of pathological pH study in this population^4^, more robust data from 122 patients demonstrated that 25% of them had abnormal postoperative pH study. More interesting is that only 8.2% flipped from normal to abnormal pH study, 25.4% flipped from abnormal to normal, 49.2% were normal before surgery and remained normal after Collis, and 17.2% were abnormal preoperatively and persisted abnormal after the procedure^9^. And even though a quarter of patients may have an abnormal pH study, the subjective evaluation demonstrated symptom control that is similar to that of patients without Collis gastroplasty^1,5^. 

 Another limitation is the need for expert collaboration during image processing. Input from the main surgeon was frequently needed. This can induce bias during image interpretation. Although this might be the case, all types of fundoplication were correctly identified before any interference, and the recurrence was described independently by the radiologist. 

## CONCLUSIONS

 Collis gastroplasty reliably produces a tubular gastric conduit resembling the native esophagus in shape and volume. 3D CT is a promising tool for evaluating the postoperative morphology of the repair and anatomical recurrences, demonstrating the durable anatomical structure of Collis gastroplasty. 

## Data Availability

The datasets generated and/or analyzed during the current study are available from the corresponding author upon reasonable request.
